# The Social Evil

**Published:** 1874-04-15

**Authors:** 


### Editorial Department.

### THE SOCIAL EVIL.

OF late, a great amount of discussion has been indulged in, both on the platform and in the press, concerning the regulation of the so-called "Social Evil." Many members of the profession, both in this country and in Europe, advocate the placing of prostitutes under legal license and inspection, with the view of limiting the prevalence of syphilitic diseases.

The end sought is certainly a desirable one, but the means hitherto employed have seemed to us wholly inadequate, if not worse than useless. It is easy to see that the first enforcement of a system of registry, inspection, and license, would include a long list of prostitutes, and many cases of disease; and that each subsequent year, for three or four years, at least, the number of both would diminish, *apparently*, not by actually lessening the number engaged in prostitution, but by increasing the number who avoid the registry by a more private mode of carrying on the business.

We have never been able to see why the medical inspection of a prostitute, once a week, should materially diminish the prevalence of syphilis, so long as she is liable to embrace disease the very next hour after her inspection, and have all the week to spread it in. If it were possible to rigidly enforce a law, that *both sexes* should be examined, and receive a certificate of health before they embrace each other, some real benefit might be obtained, so far as the spread of disease is concerned. But we believe that all legislation, founded on the idea of "regulating," instead of suppressing, an acknowledged crime, is radically wrong, and actually injurious to the community. To say that a crime always has existed, and always will exist, and therefore it is better to legally recognize it, and attempt to so regulate it as to diminish some of its consequences, is simply to adopt a process of reasoning that would lead us to excuse, and attempt to *regulate*, every crime known to man. The crime of murder has been repeated ever since Cain slew his brother, and will, probably, continue to be repeated until the dawn of the millenium. Shall we, therefore, repeal the laws for suppressing the evil, and substitute "regulation;" and have the murderer's tools inspected, and give him a certificate that they are in good order, and free from poison? We have been led to the expression of these thoughts by the following item of statistics, handed to us by a medical friend:

"The Board of Health, in St. Louis, claims to have reduced venereal disease nearly 50 per cent. by the system of licensing and regulating houses of prostitution. To prove this, they have gotten up some statistics from the hospitals *under their control*. The power to control a hospital, and regulate the records, and the admission of patients, renders it possible to have any kind of figures desired. But there is a Marine Hospital there, not under their control, and from it I have gathered the following figures, and append those of the Chicago Marine Hospital, for comparison:

PERCENTAGE OF VENEREAL CASES AMONG THE PATIENTS ADMITTED TO MARINE HOSPITAL:

| | St. Louis. | Chicago. |
|---|---|---|
| During eight months of the year, preceding the license act | 27 per ct., | 17 per ct. |
| Corresponding eight months of the year, after the license | 34 " " | 12 " " |
| Year 1872 | 23 " " | 15 " " |
| Year 1873 | 27 " " | 14 " " |

